# Medical image diagnosis based on adaptive Hybrid Quantum CNN

**DOI:** 10.1186/s12880-023-01084-5

**Published:** 2023-09-14

**Authors:** Naim Ajlouni, Adem Özyavaş, Mustafa Takaoğlu, Faruk Takaoğlu, Firas Ajlouni

**Affiliations:** 1 Faculty of Engineering, Istanbul Atlas University, 34295 Istanbul, Türkiye; 2 Faculty of Engineering, Istanbul Atlas University, Hamidiye, Anadolu Cd. No:40, 34408, 34403 Kağıthane Istanbul, Turkey; 3 Tübitak Bilgem, Barış, 1802. Sk. No:1, 41400 Gebze Kocaeli, Turkey; 4 Lancashire College of Further Education, Appleby Street, Lancashire, BB1 3BL Blackburn UK; 5 The Scientific and Technological Research Council of Türkiye (TÜBİTAK), BİLGEM, Kocaeli, Türkiye; 6 Department of Computer Science, Lancashire College of Further Education, Accrington, BB5 OHJ UK

**Keywords:** Neural networks, CNN, Parameterized Quantum Circuits ‘PQC’, Hybrid QCNN, Adaptive momentum, Medical diagnosis

## Abstract

Hybrid quantum systems have shown promise in image classification by combining the strengths of both classical and quantum algorithms. These systems leverage the parallel processing power of quantum computers to perform complex computations while utilizing classical algorithms to handle the vast amounts of data involved in imaging. The hybrid approach is intended to improve accuracy and speed compared to traditional classical methods. Further research and development in this area can revolutionize the way medical images are classified and help improve patient diagnosis and treatment. The use of Conventional Neural Networks (CNN) for the classification and diagnosis of medical images using big datasets requires, in most cases, the use of special high-performance computing machines, which are very expensive and hard to access by most researchers. A new form of Machine Learning (ML), Quantum machine learning (QML), is being introduced as an emerging strategy to overcome this problem. A hybrid quantum–classical CNN uses both quantum and classical convolution layers designed to use a parameterized quantum circuit. This means that the computing model utilizes a quantum circuits approach to construct QML algorithms, which are then used to transform the quantum state to extract image hidden features. This computational acceleration is expected to achieve better algorithm performance than classical CNNs. This study intends to evaluate the performance of a Hybrid Quantum CNN (HQCNN) against a conventional CNN. This is followed by some optimizer modifications for both proposed and classical CNN methods to investigate the possible further improvement of their performance. The optimizer modification is based on forcing the optimizer to be directly adaptive to model accuracy. The optimizer adaptiveness is based on the development of an optimizer with a loss base adaptive momentum. Several algorithms are developed to achieve the above-mentioned goals, including CNN, QCNN, CNN with the adaptive optimizer, and QCNN with the Adaptive optimizer. The four algorithms are tested against a Kaggle brain dataset containing over 7000 samples. The test results show the hybrid quantum circuit algorithm outperformed the conventional CNN algorithm. The performance of both algorithms was further improved by using a fully adaptive SGD optimizer.

## Introduction

As medical data becomes more diverse and complex, medical experts require accurate and efficient diagnosis methods. The development of intelligent algorithms plays a crucial role in achieving both accuracy and speed in the diagnostic process [1–3]. These algorithms provide medical experts with the necessary tools to enhance their diagnoses [4, 5]. Currently, machine learning (ML) and predictive modelling techniques [6, 7] are utilized for such tasks. In this study, we aim to develop and investigate a Hybrid Quantum Convolutional Neural Network (HQCNN) as an alternative to conventional CNN-based models. Additionally, we aim to enhance the performance of the HQCNN by incorporating a fully variable adaptive momentum-based optimizer, which will improve the convergence rate of the loss function.

Quantum mechanics provides a mathematical framework and set of physical principles that researchers harness to achieve faster computational processes through quantum computing [8, 9]. Quantum computing utilizes the unique properties of quantum mechanics to perform information processing [10, 11]. Quantum technology aims to develop quantum computers with increased computational capabilities [12]. The ability of quantum models to leverage parallel computing has led to the emergence of Quantum Machine Learning (QML) approaches, with Parameterized Quantum Computing (PQC) being a popular technique. Researchers have demonstrated the enhanced performance of QML in various applications [13–16]. Notably, Peruzzo et al. introduced the variational quantum eigensolver (VQE) [17], and subsequent work by McClean et al. improved its optimization scheme [18]. Schuld et al. employed PQC to construct a quantum classifier [19], while Zeng et al. developed a Hybrid Quantum Neural Network (HQNN) using a full measurement strategy, achieving high accuracy in binary and multiclassification tasks [20]. Several QML algorithms based on PQC have been proposed [21–27], demonstrating superior performance and computational abilities compared to conventional algorithms [28–31]. Moreover, Cong et al. proposed a Quantum Convolutional Neural Network (QCNN) for phase classification and quantum error correction code optimization [32]. Li et al. introduced a Quantum Deep Convolutional Neural Network (QDCNN) that demonstrated exponential computational acceleration [33]. Parthasarathy et al. proposed a Quantum Optical Convolutional Neural Network (QOCNN) that integrated quantum convolution and quantum optics [34]. These advancements highlight the potential of quantum-inspired models in various domains. Medical images play a crucial role in diagnosing and treating a wide range of medical conditions. They provide a non-invasive way for healthcare providers to visualize internal structures and assess the extent of disease or injury. For example, X-rays, CT scans, MRIs, and ultrasound images can help diagnose broken bones, tumours, heart problems, and other conditions. They can also provide important information about the size, shape, and location of a problem, which can be used to plan the most appropriate treatment. Medical images are essential for making an accurate diagnosis and determining the best course of treatment. They are also crucial for monitoring a condition's progression and evaluating treatment effectiveness.

Medical imaging plays a crucial role in accurate diagnosis, treatment planning, and monitoring the progression of medical conditions. However, classical Convolutional Neural Networks (CNNs) face speed challenges when it comes to classifying medical images, particularly with large and high-dimensional images. Training a classical CNN on a large dataset can be time-consuming, and the testing phase can also be slow, especially for large images that require extensive computations to produce classification results. Furthermore, the use of large and complex models can result in slow inference times, which is a significant concern in real-time applications like medical imaging. These speed limitations hinder the efficient analysis and processing of medical images, potentially impacting the timely delivery of patient care and decision-making by medical professionals. As a result, there is a need for alternative approaches that can accelerate the classification process and improve the efficiency of medical image analysis.

This study aims to develop a hybrid quantum–classical model for medical image classification by combining Parameterized Quantum Computing (PQC) with classical neural networks (NN). The hybrid model harnesses the high-performance capabilities of PQC while preserving the fundamental characteristics of NN. The quantum convolutional layer, constructed using quantum convolution kernels based on PQC, processes medical images transformed into quantum states. The Quantum pooling layer performs pooling operations by measuring qubits at specific locations to obtain quantum system evolution results, which are then fed into a fully connected layer for further processing. Two different medical image datasets are used to evaluate the proposed method. Additionally, the model performance is enhanced by incorporating a fully adaptive momentum algorithm (FA_HQCNN). The performance of the FA_HQCNN algorithm will be compared against the HQCNN and a conventional CNN algorithm.

Simulation results demonstrate that the hybrid quantum circuit model surpasses the conventional CNN model by achieving optimal validation accuracy in approximately two-thirds of the number of epochs. The hybrid quantum algorithm exhibits strong learning ability and achieves high image classification accuracy. Furthermore, the utilization of the fully adaptive SGD optimizer further enhances the performance of the HQCNN.

The findings of this study support the effectiveness of the hybrid quantum–classical model for medical image classification. The combination of PQC and classical NN demonstrates improved convergence speed and classification accuracy, with the fully adaptive SGD optimizer enhancing the model's performance even further.

This paper is structured as follows: Sect. "Introduction". Introduction: Provides an overview of the motivation and background of the study. Sect. "Convolutional Neural Network", Conventional CNN: Briefly introduces the conventional Convolutional Neural Network (CNN) model, highlighting its strengths and limitations in medical image classification. Sect. "Parameterized Quantum Circuits", Parameterized Quantum Computing (PQC) Theory: Presents the theory behind Parameterized Quantum Computing, explaining how it can be used to construct quantum convolutional layers for image processing tasks. Sect. "Variable Adaptive Optimizer", Fully Adaptive Optimizer, introduces the fully adaptive optimizer used in the fully connected layer of the proposed model. Explains the algorithm and its benefits for improving the convergence and performance of the hybrid quantum CNN. Sect. "Proposed Method", Architecture of the Adaptive Hybrid Quantum CNN Algorithm: Describes the architecture and design of the proposed adaptive Hybrid Quantum CNN algorithm. Outlines the integration of PQC with classical neural networks and how the hybrid model is constructed. Sect. "Simulation and Results", Simulation and Testing Results: Presents the results of simulations and testing conducted to evaluate the performance of the proposed algorithm. Provides quantitative metrics, such as accuracy and convergence speed, to assess the effectiveness of the hybrid quantum CNN model. Section 7, Conclusion: Summarizes the main findings of the study and highlights the advantages and contributions of the proposed adaptive Hybrid Quantum CNN algorithm for medical image classification. Discusses potential future directions and areas for further improvement.

## Convolutional neural network

The Convolutional Neural Network (CNN) is a widely utilized architecture for various processing tasks, including image recognition and feature classification. It comprises three main layers: Convolution, pooling, and fully connected layers. The convolution and pooling layers play a crucial role in extracting features from input images, while the fully connected layer maps these extracted features to the output, enabling classification based on the identified features. A typical CNN structure consists of multiple blocks, incorporating convolution, pooling, and fully connected layers.

**Fig. 1:**
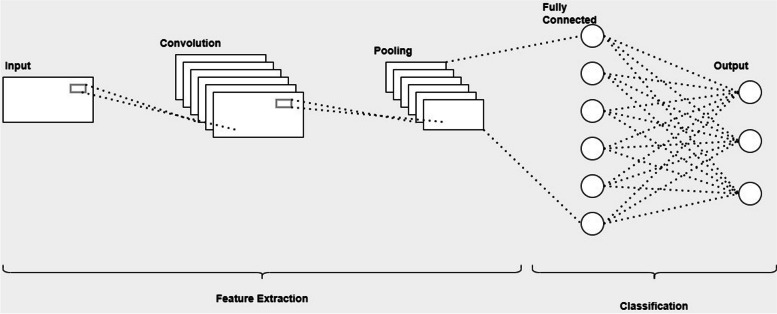
Conventional CNN structure

The Convolution layer is a key component of CNNs, as it efficiently extracts features from images by scanning each pixel and recognizing that relevant features can exist anywhere within the image. To optimize the CNN, a kernel is trained, and the discrepancy between the network's output and the ground truth labels is minimized using techniques such as Backpropagation and the selection of an appropriate optimizer. In certain cases, the CNN structure may include multiple convolution and pooling layers, followed by a fully connected layer. Figure 1 provides a representative illustration of a CNN configuration.

Overall, the CNN architecture, with its convolution, pooling, and fully connected layers, forms a powerful framework for feature extraction and classification in image processing tasks. It has proven to be highly effective in various domains, including computer vision and medical image analysis.

Within the convolution layer of a Convolutional Neural Network (CNN), the crucial process of feature extraction occurs. This layer applies a series of operations, both linear and nonlinear, to the input data.

Initially, the convolution operation takes place, where a set of learnable filters, also known as kernels or feature detectors, slide over the input data, extracting local patterns or features. Each filter performs a dot product between its weights and a small region of the input, producing a feature map. This process captures various patterns, such as edges, corners, and textures, at different spatial locations in the input.

Following the convolution operation, a nonlinear activation function is applied elementwise to the output feature map. This nonlinearity introduces flexibility and enhances the model's ability to capture complex relationships between the input and learned features. Common activation functions used in CNNs include the sigmoid function, which squashes the output between 0 and 1, and the rectified linear unit (ReLU), which sets negative values to zero and leaves positive values unchanged.

The activation function's role is to introduce nonlinearity into the network, enabling it to learn and represent more complex patterns in the data. By applying these nonlinear transformations to the linear outputs of the convolution operation, the convolution layer becomes capable of capturing rich and abstract features from the input data. Overall, the convolution layer's combination of linear and nonlinear operations, along with the activation function, allows CNNs to effectively extract and represent meaningful features from input images or data, enabling subsequent layers to learn higher-level representations and perform accurate classification or regression tasks.

After the convolution layer, the pooling layer plays a crucial role in downsampling the extracted features and reducing their dimensionality. This downsampling process helps achieve translation invariance, making the network more robust to small shifts and distortions in the input data. The pooling layer divides the input feature maps into non-overlapping regions, often referred to as pooling windows or pooling kernels. Within each pooling window, a pooling operation is applied to summarize the information. Two common types of pooling operations are max pooling and average pooling. In the case of max pooling, the maximum value within each pooling window is extracted as the representative value for that region. This means that only the most prominent feature in each region is retained, discarding less relevant information. This process helps capture the most salient features and reduce the impact of minor variations or noise in the input.

On the other hand, average pooling computes the average value within each pooling window, providing a summary of the overall intensity or activation levels in that region. This pooling method can be useful in certain scenarios where capturing the average information across a region is more relevant than focusing solely on the maximum value.

**Fig. 2:**
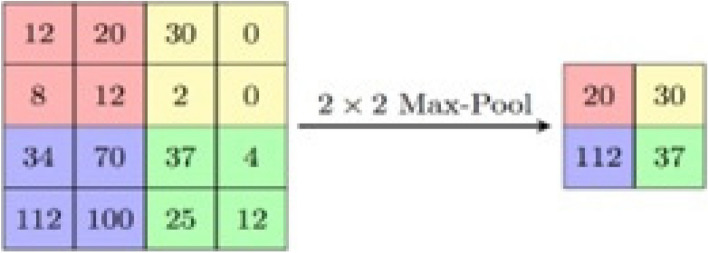
MAX pooling operation

In this study, the implementation employs max pooling, which extracts the maximum value within each pooling window. By retaining only the most significant features, max pooling helps preserve the essential information while reducing the dimensionality of the feature maps. The downsampling achieved by the pooling layer leads to a more compact representation, reducing the number of parameters and computational complexity in the subsequent layers. For a visual representation of the pooling process, you can refer to Fig. 2, which illustrates the pooling layer's operation in the overall CNN architecture.

## Parameterized quantum circuits

**Fig. 3:**
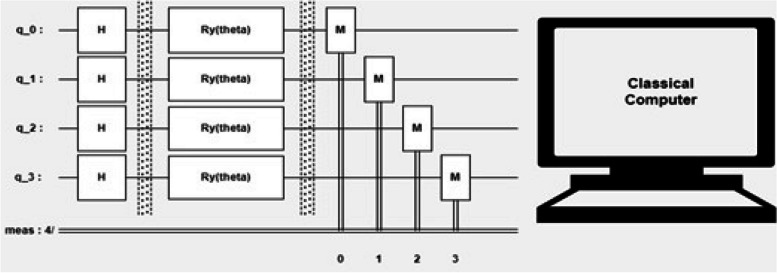
Schematic of PQC

Parameterized Quantum Circuits (PQCs) are quantum circuits that incorporate adjustable parameters, enabling the optimization of quantum computations; the Schematic of PQC is shown in Fig. 3. PQCs find extensive applications in quantum machine learning and quantum optimization since they can be trained and optimized for specific tasks. Essentially, a parameterized quantum circuit (PQC) is a type of quantum circuit where certain parameters, such as rotation angles, can be modified to achieve various objectives or enhance circuit performance. These parameters are represented by real numbers, while the quantum gates are represented by unitary matrices. By combining gates and parameters, the evolution of a quantum state is defined, creating a quantum circuit that can be optimized using classical algorithms. The goal of optimization is to determine parameter values that minimize a cost function, which evaluates the quality of the circuit's output. PQCs have found utility in several quantum computing tasks, including quantum machine learning, quantum optimization, and quantum error correction. The flexibility to adjust parameters makes PQCs versatile and allows customization for specific tasks, making them invaluable in the field of quantum computing. In a PQC, the quantum gates are parameterized, implying that a set of parameters controls the unitary operations applied to quantum states. These parameters are then optimized to minimize a cost function, which quantifies the disparity between the desired and actual outputs of the quantum circuit. Classical optimization algorithms like Gradient Descent (GD) or Adam are utilized for this optimization process. For this study, the aim is to develop an adaptive optimizer based on Stochastic Gradient Descent (SGD), which can adjust its variables according to specific requirements. The key advantage of PQCs is that they allow for the flexible and efficient control of quantum states, which is important for tasks such as quantum image classification, quantum state preparation, and quantum optimization. Additionally, PQCs can be combined with classical machine learning algorithms, such as neural networks, to create hybrid quantum–classical systems that can leverage the strengths of both quantum and classical computing. The parametric gate includes a single qubit rotation gate, represented by:

1 $$\begin{array}{l}R_x\left(\theta\right)=\begin{bmatrix}\cos\left(\frac\theta2\right)&-i\;\sin\left(\frac\theta2\right)\\-i\;\sin\left(\frac\theta2\right)&\cos\left(\frac\theta2\right)\end{bmatrix},\\R_y\left(\theta\right)=\begin{bmatrix}\cos\left(\frac\theta2\right)&-\sin\left(\frac\theta2\right)\\\sin\left(\frac\theta2\right)&\cos\left(\frac\theta2\right)\end{bmatrix},\\R_z\left(\theta\right)=\begin{bmatrix}\begin{array}{lc}e^{-i\left(\frac\theta2\right)}&0\\0&e^{i\left(\frac\theta2\right)}\end{array}\end{bmatrix},\end{array}$$

A quantum circuit $U(w)$ is utilized to apply a unitary transformation to a quantum state $|x\rangle$, resulting in the production of a quantum output state $|y\rangle$. The transformation is represented as:

2 $$|y\rangle = U(w)|x\rangle$$

Here, $w$ represents the set of parameters associated with the quantum circuit $U$. By adjusting the values of these parameters; the quantum circuit can be optimized to achieve specific computational goals or improve its performance. The resulting output state $|y\rangle$ is the result of the unitary transformation applied to the input state $|x\rangle$ using the specified parameter values w.

In the quantum computing process, an input quantum state undergoes a unitary transformation within the quantum circuit. Subsequently, the resulting output quantum state is subjected to measurement. The measurement outcomes are then fed into a classical computer, where they are utilized to calculate a loss value. This loss value serves as a metric to evaluate the performance or effectiveness of the quantum circuit.

An iterative process involving the classical computer takes place to optimize the quantum circuit parameters. The classical computer adjusts the parameters of the quantum circuit based on the measurement results and the calculated loss value. This adjustment aims to improve the circuit's performance by iteratively refining the parameter values. The optimization process continues for multiple iterations until certain conditions are met. These conditions could include achieving a satisfactory expectation value, which signifies the desired outcome, or reaching a maximum predefined number of iterations. In summary, the quantum computer applies a unitary transformation to the input quantum state using quantum circuits, followed by measurement of the output quantum state. The classical computer then computes the loss value based on the measurement outcomes, and the quantum circuit parameters are optimized through iterative adjustments made by the classical computer until specific criteria are fulfilled or a maximum number of iterations is reached.

The quantum gate is represented by the Pauli-X matrix:

3 $$X=\begin{bmatrix}0&1\\1&0\end{bmatrix}=\vert0\rangle\langle1\vert+\vert1\rangle\langle0\vert$$

To observe the impact of a gate on a qubit state vector is multiplied by the corresponding gate. In the case of the X-gate, it switches the amplitudes of state $|0\rangle$ and $|1\rangle$. Specifically, when the X-gate is applied, the states $|0\rangle$ and $|1\rangle$ interchange their amplitudes. Mathematically, this can be represented as $X|0\rangle = |1\rangle$ and $X|1\rangle = |0\rangle$. Thus, the X-gate transforms the qubit state as $X(|0\rangle |0\rangle ) = |1\rangle |0\rangle$ and $X(|1\rangle |1\rangle ) = |0\rangle |1\rangle$. In other words, the X-gate swaps the amplitudes between the basis states $|0\rangle$ and |1⟩ within the qubit state vector. This can be written as:

4 $$\mathrm X\left|0\right.\rangle=\begin{bmatrix}0&1\\1&0\end{bmatrix}\;\begin{bmatrix}0\\1\end{bmatrix}=\begin{bmatrix}0\\1\end{bmatrix}=\vert1\rangle$$

The $Y$ and $Z$ Pauli matrices can indeed act as the $Y$ and $Z$ gates in quantum circuits. The $Y$ gate corresponds to a rotation of $\pi$ (180 degrees) around the y-axis of the Bloch sphere, while the $Z$ gate corresponds to a rotation of $\pi$ around the z-axis.

The $Y$ gate has the following effect on qubit states $Y|0\rangle = i|1\rangle$ and $Y|1\rangle = -i|0\rangle$. This gate introduces complex phases and interchanges the amplitudes of the qubit states |0⟩ and |1⟩. Similarly, the $Z$ gate transforms the qubit states as $Z|0\rangle = |0\rangle$ and $Z|1\rangle = -|1\rangle$. The $Z$ gate does not alter the state $|0\rangle$, but it flips the phase of the state $|1\rangle$ by multiplying it by -1. In summary, the $Y$ and $Z$ Pauli matrices can act as the $Y$ and $Z$ gates, respectively, in quantum circuits. The $Y$ gate performs a $\pi$ rotation around the y-axis, interchanging amplitudes and introducing complex phases. The $Z$ gate performs a $\pi$ rotation around the z-axis, leaving the $|0\rangle$ state unchanged and flipping the phase of the $|1\rangle$ state. This is achieved by Eq. 5.

5 $$\begin{aligned}Y=\begin{bmatrix}0&-i\\i&0\end{bmatrix}&Z=\begin{bmatrix}1&0\\0&-1\end{bmatrix}\\Y=-i\vert0\rangle\langle1\vert+i\vert1\rangle\langle0\vert&Z=\vert0\rangle\langle0\vert-\vert1\rangle\langle1\vert\end{aligned}$$

The U gate, known as the arbitrary single-qubit rotation gate, performs a rotation around the bloch-sphere using three Euler angles $\theta , \lambda ,$ and $\varphi$. The U operation is given as:

6 $$U=\begin{bmatrix}\cos\left(\frac\theta2\right)&-e^{i\lambda}\sin\left(\frac\theta2\right)\\e^{i\phi}\sin\left(\frac\theta2\right)&e^{i(\phi+\lambda)}cos\left(\frac\theta2\right)\end{bmatrix}$$

The U gate, also known as the arbitrary single-qubit rotation gate, can replicate all other single-qubit gates. This property is often referred to as gate universality. By appropriately choosing the three Euler angles $(\theta , \varphi , \lambda )$ in the U gate, one can reproduce the effects of other single-qubit gates such as the Pauli gates ($X, Y, Z$), the Hadamard gate (H), and many other common gates used in quantum computing. Gate universality is a significant property as it means that with a U gate and additional two-qubit gates, such as the CNOT gate, with this in mind, one can construct any quantum computation or operation. The ability of the U gate to replicate other gates allows for flexibility and versatility in designing quantum algorithms and circuits. The rotation of $\theta , \varphi ,$ and $\lambda$ by $\pi , \pi , and \frac{\pi }{2}$, achieves the replication for the X gate as:

7 $$X=U(\pi ,\pi ,\pi /2)=U(\theta ,\varphi , \lambda )$$

## Variable adaptive optimizer

The variable adaptive Gradient Descent optimizer proposed in this study aims to enhance the traditional gradient descent optimization algorithm. Gradient descent is a widely used optimization technique employed in various machine learning algorithms, including linear regression, classification, and Back Propagation (BP) neural networks. The conventional gradient descent algorithm relies on the first-order derivative of a loss function to iteratively update the weights of the model, aiming to reach a minimum. It calculates the weights based on the gradient of the loss function with respect to the weights. In the BP algorithm, the loss is propagated through the network, layer by layer, and the model's parameters (weights) are adjusted based on minimizing the accumulated losses. The original BP algorithm was introduced by reference [11]. In this case, the initial weight ${W}_{0}$ and the iterative increment formula are provided to update the weights during the optimization process. However, in the proposed study, a variable adaptive Gradient Descent optimizer is employed. This optimizer aims to improve upon the traditional gradient descent algorithm by incorporating adaptive techniques. It adapts the learning rate or step size dynamically during the optimization process to achieve faster convergence and better performance. In this case, the initial weight ${W}_{0}$, iterative increment formula is given as

8 $$\begin{array}{cc}\Delta w(n+1)=\eta\delta \_oy(n)+\alpha \Delta w(n)& n=\mathrm{0,1},\dots \end{array}$$

where $\eta > 0$ is the learning rate used to indicate distance along the gradient's negative direction. This algorithm convergence speed is slow due to the inherent behaviour of the activation function in the network, which worsens for multi-hidden layer networks. The second problem with this method is the difficulty in choosing a proper learning rate $\eta$ to achieve fast learning while maintaining the learning procedure stable. To overcome these problems, it is proposed to replace the fixed learning rate value with a suitable adaptive function directly dependent on loss change.

In the proposed approach, the fixed learning rate value ($\eta$) in the conventional gradient descent algorithm is replaced with an adaptive function that directly depends on the change in the loss. This adaptive function aims to address the slow convergence speed and the challenge of choosing an appropriate learning rate. The inherent behavior of the activation function in the network can lead to slow convergence, especially in multi-hidden layer networks. By introducing an adaptive learning rate that takes into account the change in the loss function, the proposed method seeks to improve the convergence speed. The adaptive learning rate function dynamically adjusts the learning rate based on the observed changes in the loss function. This adaptive behavior allows for faster learning while maintaining stability in the learning process. By adapting the learning rate based on the loss change, the method aims to strike a balance between learning speed and stability. The hidden layer is defined by,

9 $${H}_{j}={b}_{in}+\sum\nolimits_{i=1}^{N}{\mathbf{x}}_{i}{\mathbf{w}}_{ij}$$

where ${b}_{in}$ is a bias input layer, the hidden layer will pass through the activation function $(f)$. In this study, the SoftMax function is used. The SoftMax activation function is represented as

10 $$P(y=j|x)=\frac{{e}^{x{w}_{j}}}{\sum_{i=1}^{K}{e}^{x{w}_{j}}}$$

After calculating the overall output by multiplying the output of the hidden layer neurons with the hidden layer weights ${\mathbf{w}}_{jk}$, the results then, pass through a sigmoid function (called threshold) as shown below.

11 $${y}_{k}={b}_{n}+\sum\nolimits_{j=1}^{m}{\mathbf{w}}_{jk}P(y)$$

where ${b}_{n}$ is the bias of the hidden layer and $k$ output neurons.

The network error equation is given by $E$ for each pattern ($p$) it is calculated by subtracting the overall output $\mathrm{o}$ from the target $t$;

12 $$E=\frac{1}{2}\sum\nolimits_{j=1}^{p}{\left({t}_{j}-{o}_{j}\right)}^{2}$$

The weight update equation with both learning and momentum terms is given as:

13 $${\Delta \mathbf{w}}_{ji}\left(n+1\right)={\eta\delta }_{y}{\mathbf{x}}_{i}(n)+\alpha {\Delta \mathbf{w}}_{ji}(n)$$

14 $$\begin{array}{cc}{\Delta \mathbf{w}}_{kj}\left(n+1\right)={\eta\delta }_{o}{y}_{i}\left(n\right)+\alpha {\Delta \mathbf{w}}_{kj}\left(n\right)&\;n=\mathrm{0,1},\dots \end{array}$$

where $n$ is the iteration number, $\eta$ is the learning rate, and $\alpha$ is the momentum term. The bias update equation is given as follows:

15 $${b}_{j}\left(n\right)={b}_{j}\left(n\right)+\eta.f\left({H}_{j}\right)\left(1-f\left({H}_{j}\right)\right){\mathbf{x}}_{i}\left(n\right){\mathbf{w}}_{j}\left(n\right)e\left(n\right)$$

In this study, the variable adaptive momentum equation is represented as:

16 $$\alpha (n)=\frac{\lambda }{1-({e}^{-\left(error\left(t\right)+error\left(t-1\right)\right)})}$$

Where $\lambda <\frac{2-2\beta }{max\;eigen\;value\;of\;{\mathbf{R}}_{\mathbf{x}\mathbf{x}}}$ and the $\beta$ is the forgetting factor $(0 "\beta <1)$,

Here, $\alpha (n)$ denotes the adaptive momentum term at iteration $n$, $\lambda$ represents a constant value, $error(t)$ refers to the error or loss at the current iteration t, and $error(t-1)$ represents the error or loss at the previous iteration $t-1$.

The equation calculates the value of the adaptive momentum term based on the current and previous error values. By summing the error values and applying an exponential function, the equation incorporates information from past iterations to influence the momentum term. The learning rate parameter $\eta$ usually controls the adaptive momentum term $\alpha$. In this case, the learning rate $\eta$ is indirectly affected by the adaptive momentum through the equation's formulation. The value of $\alpha (n)$ obtained from the equation impacts the momentum, which in turn influences the learning rate and the optimization process. The purpose of the variable adaptive momentum equation is to adjust the momentum term dynamically based on the error values. By incorporating the past errors, the equation aims to adaptively update the momentum to improve convergence and achieve lower performance error. The initial values of $\beta$ in the variable adaptive momentum equation are set to be sufficiently large. Consequently, the term $\lambda$ approaches unity, resulting in the initial value of $\alpha (n)$ being relatively large. With this information, Eqs. (12) and (13) can be rewritten as follows:

17 $${\Delta \mathbf{w}}_{ji}\left(n+1\right)=\eta {\delta }_{y}{\mathbf{x}}_{i}(n)+\left(\frac{\lambda }{1-({e}^{-\left(E\left(t\right)+E\left(t-1\right)\right)})}\right){\Delta \mathbf{w}}_{ji}(n)$$

18 $${\Delta\mathbf w}_{ji}\left(n+1\right)=\eta\delta_y{\mathbf x}_i(n)+\left(\frac\lambda{1-(e^{-\left(E\left(t\right)+E\left(t-1\right)\right)})}\right){\Delta\mathbf w}_{ji}(n),\;n=0,1,\dots$$

where $n$ represents the number of iterations, and $\Delta \mathbf{w}$ is defined as the updating weights.

In Eq. 16, $\alpha (n)$ represents the adaptive momentum term at iteration $n$, $\alpha (n-1)$ is the previous value of the adaptive momentum term, $\beta$ represents a constant, $error(t)$ denotes the error or loss at the current iteration $t$, and $error(t-1)$ represents the error or loss at the previous iteration $t-1$. The equations show that the current value of the adaptive momentum $\alpha (n)$ is updated based on the previous value $\alpha (n-1)$, and the learning rate $\eta (n)$ is updated based on the initial learning rate η. By considering the initial large values of $\beta$, the equation ensures that the initial $\alpha (n)$ and $\eta (n)$ values are relatively large, facilitating exploration in the early stages of the optimization. As the optimization progresses and the error values decrease, the exponential term gradually diminishes, leading to a reduction in $\alpha (n)$ values and allowing for finer adjustments towards convergence.

In this study, a custom learning rate schedular with momentum based on the loss values during the training is designed. The custom learning rate schedular decays the learning rate at each epoch. The schedular is called during training; at each epoch, it evaluates the model's loss on the test set and stores it in the `loss_list’, calculating the momentum for the current epoch based on the loss history using the `calculate_momentum` function. Update the learning rate using the custom learning rate scheduler defined by the `custom_lr_scheduler` function. Perform model training with the current learning rate and momentum using the SGD optimizer. In this study, this sequence is defined as a fully adaptive SGD optimizer. Table 1, shows the pseudo code for the fully adaptive SGD optimizer.

**Table 1:** Pseudo-code for a fully adaptive SGD optimizer

# Pseudo-code for Custom Learning Rate Scheduler with Momentum Calculation
# Step 1: Define the custom learning rate scheduler
def custom_lr_scheduler(epoch, initial_lr = 0.01):
lr_decay = 0.1
lr = initial_lr * lr_decay ** epoch
return lr
# Step 2: Define a function to calculate momentum based on loss history
def calculate_momentum(loss_list, current_epoch):
if current_epoch > = 2:
loss_t_minus_1 = loss_list[current_epoch—2]
loss_t = loss_list[current_epoch—1]
beta = 1.0
momentum = beta / (1.0 – np.exp(—(loss_t + loss_t_minus_1))
# Optionally, you can set a maximum momentum value (e.g., 0.999) if necessary
# momentum = min(momentum, 0.999)
else:
# Set a default momentum value for the first two epochs
momentum = 0.9
return momentum
# Step 3: Initialize the list to store loss values
init_loss = 0.0
loss_list = [init_loss]
# Step 4: Create the model and other necessary variables (not shown in the provided code)
# Step 5: Main training loop with epochs
for epoch in range(epochs):
# Step 5.1: Evaluate the model's loss on the test set and store it in the loss_list
loss = model.evaluate(x_test, y_test_cat)[0]
loss_list.append(loss)
# Step 5.2: Calculate the momentum for the current epoch based on loss history
momentum = calculate_momentum(loss_list, epoch)
# Step 5.3: Update the learning rate using the custom learning rate scheduler
learning_rate = custom_lr_scheduler(epoch)
# Step 5.5: Perform model training with the current learning rate and momentum
optimizer = SGD(lr = learning_rate, momentum = momentum, nesterov = False)
model.compile(optimizer = optimizer, loss = 'categorical_crossentropy', metrics = ['accuracy'])
model.fit(x_train, y_train_cat, epochs = 1, batch_size = batch_size)

## Proposed method

**Fig. 4:**
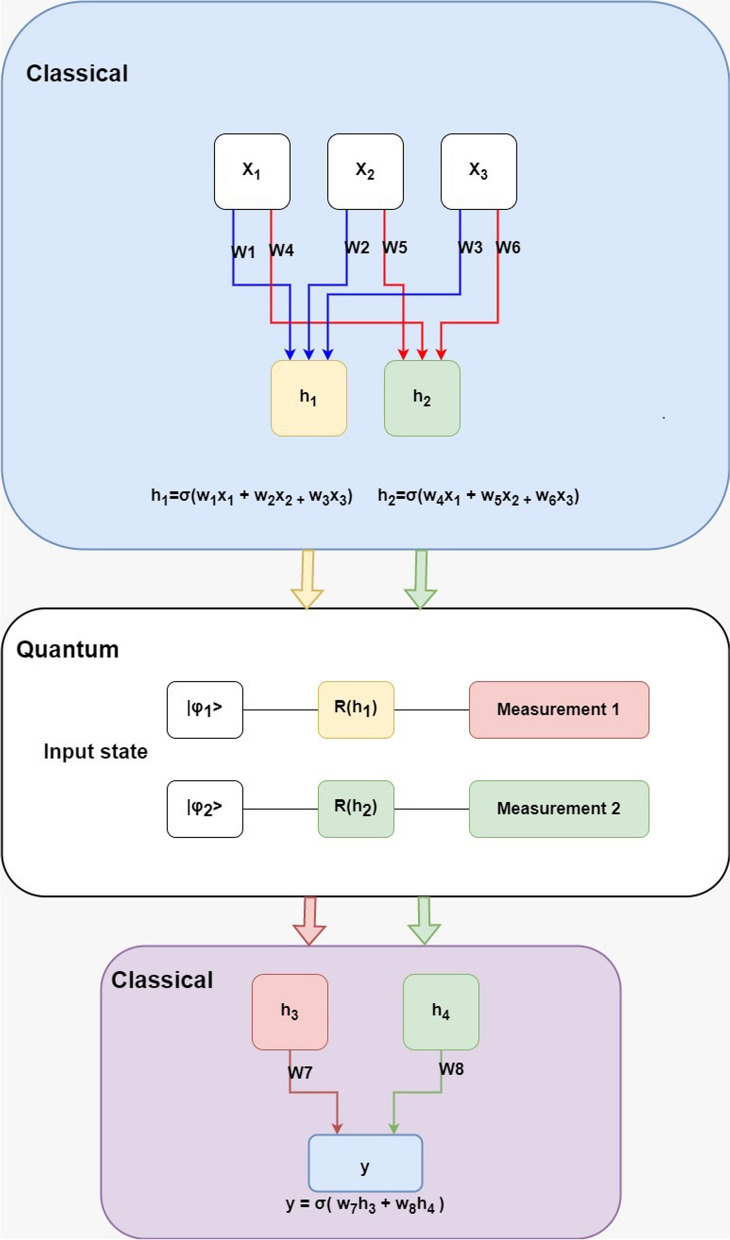
bock structure of HQCNN

**Fig. 5:**
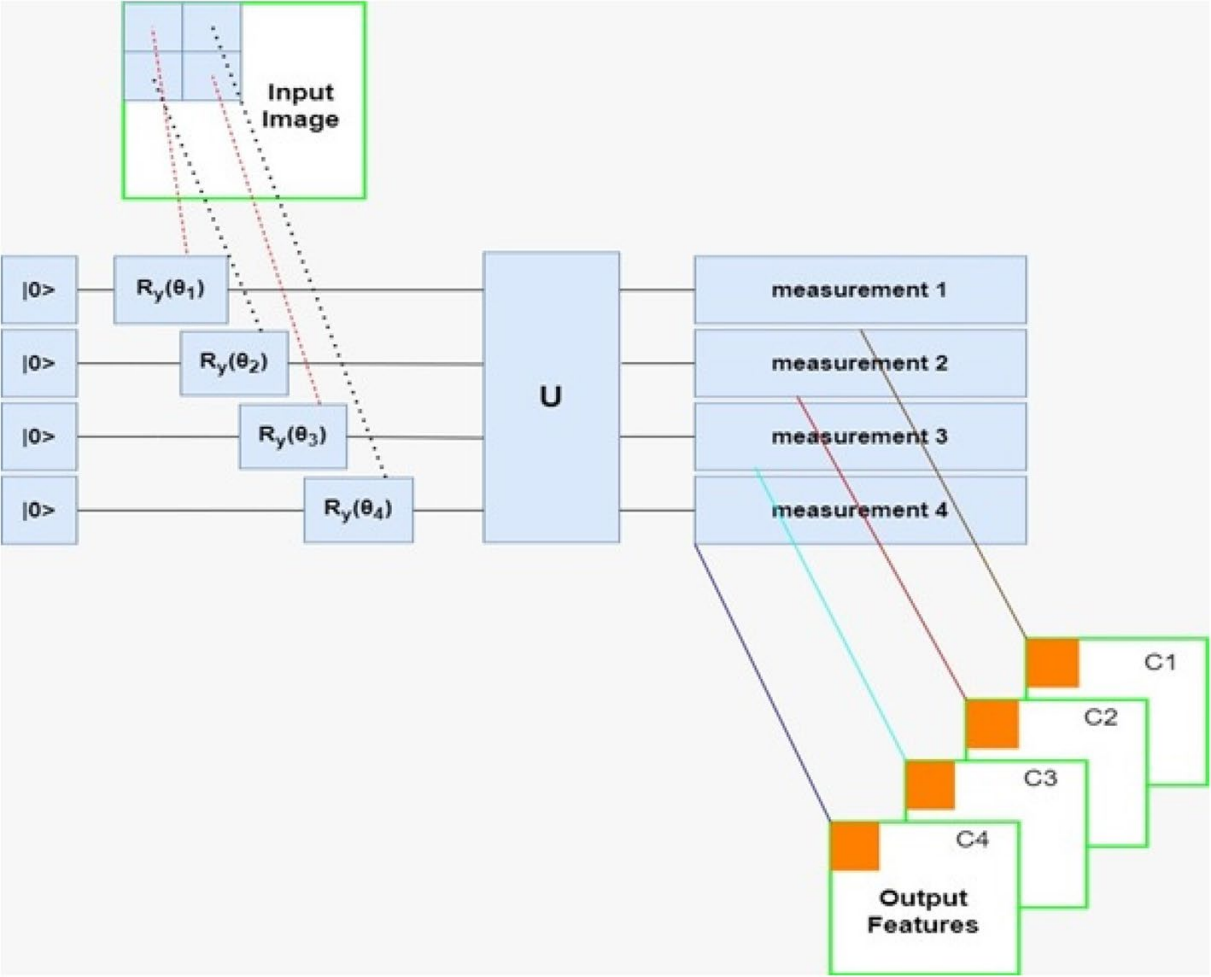
Single quantum layer image processing scheme

To overcome the big data processing speed challenges, many researchers are exploring techniques such as model compression, pruning, and hardware acceleration to make Convolutional Neural Networks (CNN) faster and more efficient. Therefore, in this study, it is proposed to use Hybrid Quantum CNN (HQCNN) to classify medical images. The proposed HQCNN will have several advantages over classical CNNs when dealing with large datasets containing high-dimensional images. The advantages include Speed: Quantum algorithms can perform some tasks exponentially faster than classical algorithms, which can result in faster processing times for large and high-dimensional images. High-dimensional feature representation: Medical images often have high-dimensional features, and quantum algorithms can provide a more compact representation of these features, reducing the complexity of the model and improving its accuracy. Improved accuracy: Quantum algorithms can be more effective at handling complex and nonlinear relationships in data, leading to improved accuracy in medical image classification. Robustness to noise: Quantum algorithms are generally more robust to noise and errors. This is important for medical image analysis, where image quality can be degraded by factors such as patient movement. To create a quantum–classical neural network, it is intended to create a hidden neural network layer using a parameterized quantum circuit. By "parameterized quantum circuit", we mean a quantum circuit where the rotation angles for each gate are specified by the components of a classical input vector; the outputs from the neural network's previous layer will be collected and used as the inputs for the parameterized circuit. The measurement statistics of the quantum circuit can then be collected and used as inputs for the following layer. A simple example is shown in Fig. 4:

In the proposed algorithm, if the input is an image, small local regions are sequentially processed with the same kernel. The results obtained for each region are usually associated with different channels of a single output pixel. The union of all the output pixels produces a new image-like object, which can be further processed by additional layers, as shown in Fig. 5.

The schematic diagram shown in Fig. 5 is a representation of a small region of a medical image which is processed by taking a small region of the input image; in our example, a $2\times 2$ square is embedded into a quantum circuit. In this case, this is achieved with parameterized rotations applied to the qubits initialized in the ground state. In this case.

**Fig. 6:**
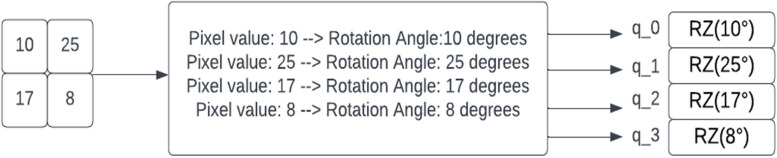
Embedding a 2 × 2 image region into a quantum circuit

**Fig. 7:**
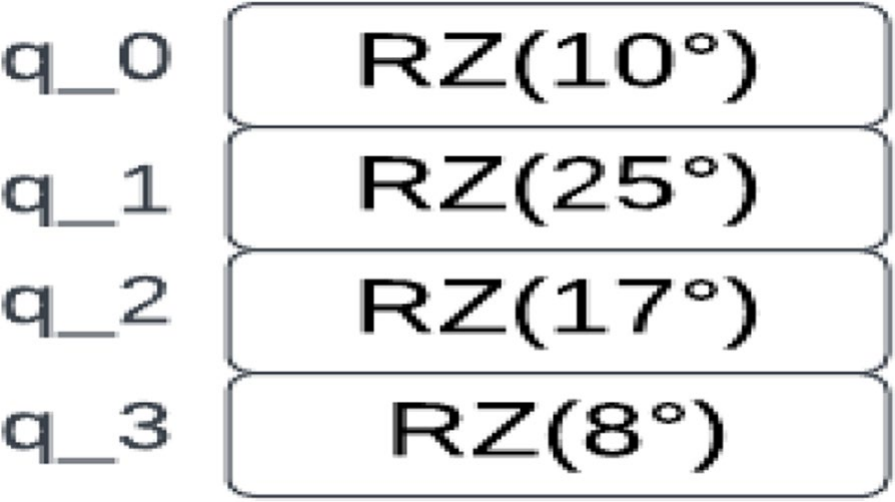
4 qubits with parameterized rotation gates

**Fig. 8:**
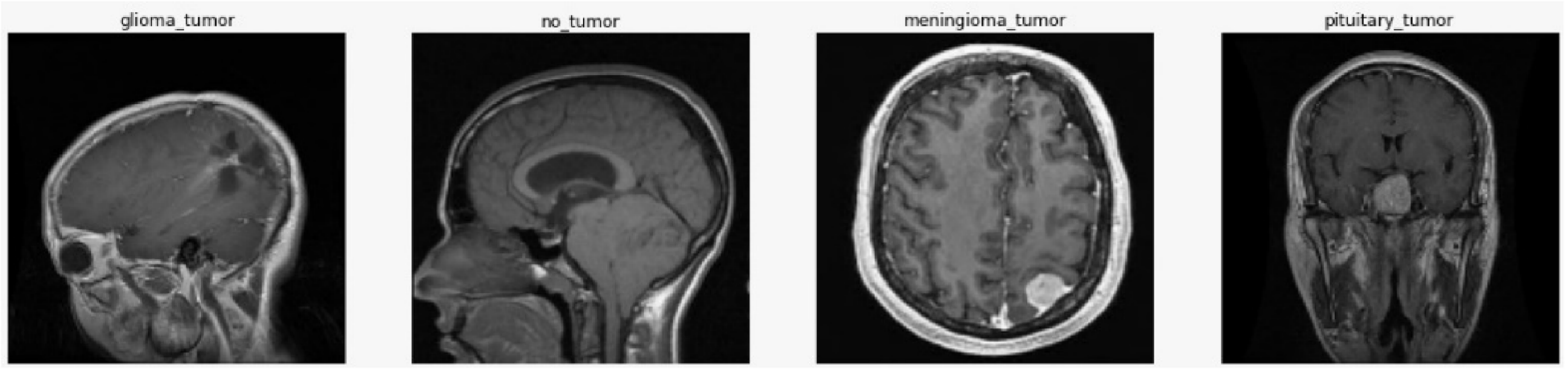
Sample from Kaggle brain dataset

Consider the image 2 × 2 square regions shown in Fig. 5 as an input to a quantum circuit. To embed this region into a quantum circuit, you can represent each pixel value as a parameter for a quantum rotation gate applied to a corresponding qubit initialized in the ground state (|0⟩ state). Figure 6 shows that the pixel values in the regions are mapped to quantum rotation angles producing a corresponding qubit. This is followed by creating a quantum circuit with the four qubits, and for each qubit, a parameterized rotation gate using the corresponding rotation angle is applied, as in Figs. 7 and 8.

In this representation, each qubit is associated with one pixel in the 2 × 2 square region. The RZ gates with angles in degrees represent the parameterized rotations applied to the qubits. These rotations encode the pixel values from the input image into the quantum circuit.

A quantum computation associated with a unitary $U$ gate is performed on the system. The unitary could be generated by a variational quantum circuit or, more simply, by a random circuit. To visualize the concept of a quantum computation associated with a unitary gate, consider a simple quantum system with two qubits (quantum bits), denoted as |q0⟩ and |q1⟩. In quantum computing, a quantum state can be represented as a vector in a complex vector space. For a two-qubit system, the quantum state can be represented as:

19 $$|\psi \rangle = \alpha |00\rangle + \beta |01\rangle + \gamma |10\rangle + \delta |11\rangle$$

Here, $\alpha , \beta , \gamma ,$ and $\delta$ are complex numbers representing the probability amplitudes of different basis states. For simplicity, let's assume the quantum state is in the following form:

20 $$|\psi \rangle = \alpha |00\rangle + \beta |01\rangle$$

Now, we want to perform a quantum computation associated with a unitary gate U on this quantum state. The unitary gate U is represented by a $2x2$ unitary matrix, and it transforms the quantum state according to the following equation:

21 $$U|\psi \rangle = U(\alpha |00\rangle + \beta |01\rangle )$$

**Fig. 9:**
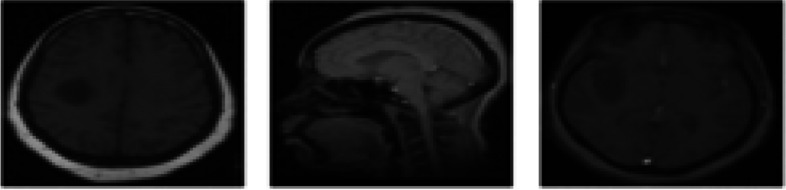
Samples from the REMBRANDT dataset

Let's consider two scenarios: one where the unitary $U$ is generated by a variational quantum circuit and the other where it is generated by a random circuit. In this case, the unitary $U$ is generated by a parameterized quantum circuit. A variational quantum circuit is a circuit with adjustable parameters that can be optimized to achieve specific objectives. In the circuit shown in Fig. 9, "Rot(θ)" and "Rot(φ)" represent single-qubit rotations parameterized by angles θ and φ, respectively. These angles can be adjusted during the optimization process. The quantum system is finally measured, obtaining a list of classical expectation values. The measurement results directly use the raw expectation values. The measurement process collapses the quantum state into one of its basis states with certain probabilities. The measurement outcomes are the classical results. The measurement results directly provide the classical outcomes, which can be used to calculate classical expectation values. For each measurement, we can compute the expectation value of a specific observable. These expectation values are the final results that can be used for various purposes, such as analyzing the behavior of a quantum algorithm or solving specific problems. Analogously to a classical convolution layer, each expectation value is mapped to a different channel of a single output pixel. The same procedure is iterated over different regions to scan the full input image, producing an output object which will be structured as a multi-channel image. The quantum convolution layers are followed by an optimization layer.

Therefore, the main distinction from classical convolution is that a quantum circuit has the capability to generate highly complex kernels that may be computationally infeasible using classical methods. The process starts by preparing the quantum state. When working with a limited number of qubits in a quantum system to address classical problems, it is often necessary to perform dimensionality reduction on the classical data. In this specific work, the image is downsampled to an $m\times m$ size, where the pixel values are scaled to the range $[\mathrm{0,1}]$. The downsampled image is then flattened into a $1\times {m}^{2}$ vector denoted as $x = [x\_1, x\_2, ..., x\_{m}^{2})]$. To convert this flattened vector $x$ into angle information $\alpha$, the operation $\alpha = \pi x$ is performed. Here, α is represented as $\alpha = [\alpha \_1, \alpha \_2, ..., \alpha \_\_{m}^{2}]$, where each $\alpha \_i$ corresponds to an angle. The angle information α is used as the rotation angle for the rotation gate $Ry$. The rotation gate $Ry$ is applied to the initial quantum state, $|0\rangle \_1\otimes|0\rangle \_2\otimes...\otimes|0\rangle \_{m}^{2})$, for encoding in an ${m}^{2}$ input quantum system. The resulting quantum state is denoted as $|\varphi \_img\rangle$. Thus, a total of $m \times m$ qubits are required to encode the down-sampled image of size $m \times m$, and the quantum state $|\varphi \_img\rangle$ is obtained by encoding all the pixels of the downsampled image. Once the quantum state $|\varphi \_img\rangle$ is obtained, the designed quantum convolution kernel $u(\theta )$, which is parameterized by four training parameters ($\theta = \theta \_1, \theta \_2, \theta \_3, \theta \_4$), is used to perform a unitary transformation on $|\varphi \_img\rangle$. This unitary transformation applies the convolutional operation using the quantum convolution kernel.

In Fig. 5, the depicted circuit represents a convolutional layer circuit with an image size of $2 \times 3$. The convolutional layer applies a unitary transformation using a convolution kernel on the qubits that correspond to the convolution window. It is important to note that the quantum convolution window aligns with the classical convolution window, but in this quantum context, it corresponds to four qubits. This means that the convolution operation is performed on a $2 \times 2$ quantum window (four qubits) within the larger quantum circuit. The purpose of repeatedly applying the convolution window on the four qubits is to retain the essential characteristics of classical convolution and extract hidden information from the quantum state. By performing this quantum convolution operation, the circuit aims to capture and process features present in the quantum state that are relevant for subsequent computations or analysis.

The quantum pooling layer operates in a similar manner to the convolution window in terms of its position within the circuit. After the pooling operation, the convolution result of each convolution window is mapped to a specific qubit. Consequently, only that particular qubit is measured to obtain the desired expectation value.

In classical convolutional neural networks (CNNs), nonlinearity is introduced through nonlinear activation functions. However, in a quantum system, nonlinearity is achieved through measurement. Once the quantum system has evolved to the desired quantum state, denoted as $|\varphi \_out\rangle$, a Z-based measurement is performed on this state to obtain the expectation value. The expectation value E is calculated as follows:

22 $$E=\langle {\varphi }_{img}|{U^{\dag}}\left(\uptheta \right){V^{\dag}}\left({Z}_{1},\dots \dots .,{Z}_{N}\right)\mathrm{VU}(\uptheta ) |{\varphi }_{out}\rangle$$

Here, ($Z\_1, ..., Z\_N$) represents a vector of $Z$ operators acting on different qubits. $V$ is a parameter-free unitary gate used in the pooling layer. $U(\theta )$ represents the product of convolution kernels $u\_i(\theta )$, where $i$ ranges from 1 to $l$. In a convolutional layer with an $m \times m$ image, $l$ equals$(m - 1{)}^{2}$, indicating the number of convolutions performed. Similarly, the pooling unit is also performed $(m - 1{)}^{2}$ times in the pooling layer. Directly measuring the output of the quantum convolutional layers yields a quantum output $E$ with dimensions $1 \times {m}^{2}$. On the other hand, measuring the output of the quantum pooling layer produces a vector $E$ with dimensions $1 \times (m - 1{)}^{2}$. This vector $E$ consists of $Z$ expectation values from different qubits. As it is not directly associated with the image label, $E$ should be passed as input to the classical fully connected layer for further processing and subsequent classification. In summary, the expectation values obtained through quantum measurements serve as the output of the quantum convolutional and pooling layers. These values are subsequently processed in the classical fully connected layer to further analyze and classify the image.

The proposed HQCNN (Hybrid Quantum Classical Convolutional Neural Network) architecture comprises three main components: a quantum convolutional layer, a quantum pooling layer, and a classical fully connected layer.

Figure 4 illustrates the structure of the HQCNN. The quantum convolutional layer consists of multiple convolution kernels designed for quantum convolution operations. These kernels perform quantum convolutions to extract relevant features from the input image, producing a feature map. The convolution operation in the quantum convolutional layer is executed using quantum circuits, taking advantage of the quantum properties to perform complex computations that may be challenging for classical approaches. The quantum pooling layer follows the quantum convolutional layer and aims to reduce the dimensionality of the convolution results. Similar to classical pooling layers, the quantum pooling layer performs pooling operations but in a quantum context. After the pooling operation, specific qubits are measured, and the measurement results are obtained. The measurement results from the quantum pooling layer are passed as inputs to the classical fully connected layer. The classical fully connected layer is a standard component in classical neural networks, responsible for processing the inputs and producing the final classification output. In this study, the Variable Adaptive Gradient Descent (VAGD) optimizer is utilized to optimize the parameters of the fully connected layer. The VAGD optimizer is a variation of the conventional Gradient Descent (GD) optimizer. It is designed to achieve faster convergence of the loss function, enabling quicker and more efficient training of the network compared to traditional optimizers.

## Simulation and results

In the study, several tests were conducted to compare the advantages of using quantum circuits in the prediction and classification of brain cancer. To be able to obtain optimal results for both the proposed HQCNN and the classical CNN models, the model’s structure must be optimal. Therefore in this study, an optimal structure for both models is obtained using Genetic Algorithms optimization for optimizing both model structures with the use of a small portion of one of the datasets as described in the Experimental section later on in this study.

### Experimental design

The experiments carried out in this work are designed to highlight the novelties introduced by the HQCNN algorithm: the generalizability of HQCNN layers compared to a classical CNN architecture, the ability to use this hybrid quantum algorithm on practical datasets, and the potential use of features introduced by the quantum circuit transformations. The experiment in this work is based on integrating quantum feature detection into a more complex neural network architecture, as the QNN framework introduces models containing nonlinearities. In this section, we will define the testing and the performance comparison of the different algorithm structures CNN, HQCNN, and the fully adaptive CNN and HQCNN.

#### Optimal CNN structure

The determination of the optimal number of layers in the CNN was achieved through a systematic approach involving training the CNN model with varying numbers of layers and filters. Once the ideal number of layers was identified, it was utilized to train the final model for performance comparison against the Hybrid Quantum CNN (HQCNN). This testing methodology was adopted due to the nonlinear relationship between the number of layers and accuracy in CNNs. While increasing the depth (number of layers) can improve performance in certain cases, it is not guaranteed, and various factors come into play. The complexity of the dataset and the problem being solved play significant roles in determining the optimal depth of the CNN. For intricate datasets, deeper CNNs might capture more intricate patterns, resulting in higher accuracy. However, for simpler datasets, a shallower CNN might already be sufficient. It's crucial to consider that adding more layers can also increase the risk of overfitting, especially with limited data. Additionally, deeper CNNs require more parameters, leading to increased memory and computational requirements. Training such models can be time-consuming and resource intensive. The challenge of vanishing and exploding gradients is also prevalent in deep networks, making it difficult to train them effectively. Although techniques like batch normalization and skip connections can mitigate this issue to some extent, it may still persist. In such cases, researchers often explore other avenues for improving accuracy without unconditionally increasing the depth of the CNN. Techniques such as transfer learning, model ensembling, and architectural innovations have been employed to enhance performance while managing computational complexity. The pseudo-code presented in Table 2 outlines an optimization process that utilizes a Genetic Algorithm (GA) to identify the most suitable hyperparameters for the CNN algorithm To identify the optimal CNN model structure. Each chromosome in the population `pop` represents a potential CNN structure, with the first element denoting the number of layers. The subsequent elements represent the number of filters and their sizes for each layer. By using the `CNN model` function with the specified parameters, the CNN architecture is constructed accordingly. The Genetic Algorithm not only searches for the optimal number of filters and filter sizes but also optimizes the number of layers, thus enhancing the CNN's performance for the specific task. In the proposed method, the testing process is split into two sections. Initially, a portion of the dataset is utilized to evaluate and determine the optimal CNN structure. Subsequently, the CNN model is trained using the entire dataset with the identified optimal structure.

**Table 2:** GA code snippet for defining Optimal CNN structure

def fitness(pop, X, y, epochs):
pop_accuracy = []
for i in range(len(pop)):
num_layers = pop[i][0]
n_filters = pop[i][1:1 + num_layers]
s_filters = pop[i][1 + num_layers:]
model = cnn_model(num_layers, n_filters, s_filters)
k = model.fit(X, y, batch_size = 32, epochs = epochs)
accuracy = k.history["accuracy"]
pop_accuracy.append(max(accuracy))
return pop_accuracy

Table 3, illustrates the resulting optimal CNN structure.

**Table 3:** Optimal CNN structure

Layer	Filter	Filter size	Stride	Total Parameters
Convolution	32	2 × 2x32	1	2048
Max Pool		2 × 2	2	0
Convolution	64	2 × 2x64	1	16,384
Max Pool		2 × 2	2	0
Convolution	64	2 × 2x64	1	16,384
Max Pool		2 × 2	2	0
Flatten	28,800	1 × 1x512	1	0
Dense	512	1 × 1x512	1	14,745,600
Dense	4	1 × 1x4	1	2048

The total number of parameters in the above CNN structure is 14,751,912. This structure is used to train and test the CNN model against both datasets.

#### Defining optimal Hybrid Quantum CNN Structure

In this study, it is intended to design an optimal HQCNN algorithm for the classification of DICOM medical type images. For a HQCNN algorithm to be optimal it requires to have the best hyperparameters (number of circuits, circuit depth, and learning rate). To achieve the required goal, Genetic Algorithms (GA) is utilized to obtain the best hyperparameters. The pseudo-code presented in Table 4, outlines an optimization process that utilizes a Genetic Algorithm (GA) to identify the most suitable hyperparameters for the HQCNN algorithm. This process involves working through a population of hyperparameter combinations to achieve the best-performing model for a given dataset of DICOM medical images. First, a portion of the DICOM dataset is loaded to enhance efficiency. The data is then divided into two sets: one for training the model and another for evaluating its performance. To assess the performance of the HQCNN model with different hyperparameter combinations, a fitness function is defined. This function measures how well the model performs based on these specific configurations. The GA begins by creating a random initial population of individuals, each representing a distinct set of hyperparameters for the HQCNN model. The GA evaluation loop is then executed, where the fitness of the current individuals in the population is assessed. Based on their fitness scores, certain individuals are selected to produce the next generation, which involves applying genetic operations like crossover and mutation. This evaluation loop is repeated for multiple generations, allowing the GA to continually refine the population and explore different hyperparameter combinations. Finally, after the evolutionary loop, the best individuals are identified based on their fitness scores. These individuals correspond to the optimal hyperparameter combinations that yield the highest performance for the HQCNN algorithm.

**Table 4:** Pseudo-code for defining the best HQCNN algorithm hyperparameters

# Pseudo-code for Hyperparameter Optimization using Genetic Algorithm
# Step 1: Load DICOM medical images and labels
X, y = load_medical_images_and_labels()
# Step 2: Split the dataset into training and test sets
X_train, X_test, y_train, y_test = train_test_split(X, y, test_size = 0.2, random_state = 42)
# Step 3: Define the evaluation function (fitness function)
def evaluate_model(individual):
num_circuits, circuit_depth, learning_rate = individual
model = create_model_with_hyperparameters(num_circuits, circuit_depth, learning_rate)
# Train the model on the training set
model.fit(X_train, y_train, epochs = 5, batch_size = 32, verbose = 0)
# Evaluate the model on the test set
y_pred = model.predict_classes(X_test)
accuracy = accuracy_score(y_test, y_pred)
# Get the number of parameters in the model
num_params = model.count_params()
return accuracy, -num_params # Maximizing accuracy and minimizing number of parameters
# Step 4: Initialize the Genetic Algorithm
population = initialize_population()
# Step 5: Main evolutionary loop
for gen in range(NUM_GENERATIONS):
offspring = create_offspring(population)
fitness_values = evaluate_fitness(offspring)
assign_fitness_values_to_individuals(offspring, fitness_values)
population = select_next_generation(offspring)
# Step 6: Get the best individual from the final population
best_individual = select_best_individual(population)
# Step 7: Print the best hyperparameters and the corresponding accuracy
best_num_circuits, best_circuit_depth, best_learning_rate = best_individual
best_accuracy, best_num_params = evaluate_model(best_individual)

The optimized parameters for the HQCNN found by the GA optimization are the number of Quantum circuits is four, the number of filters is three, and the number of qubits is four. The optimal architecture of the hybrid quantum convolutional neural network obtained by the GA is defined as one quantum convolutional layer, one pooling layer, one flattened layer, and three dense layers with sizes 128, 64, and 4. Therefore, the total number of trainable variables for the entire model is 8256. Table 5, illustrates the resulting optimal HQCNN structure.

**Table 5:** Optimal HQCNN structure

Layer	Unary gate	Number of qubits	trainable variables
Quantum Convolutional Layer	1	4	4
Pooling Layer			0
Flatten Layer			0
First Dense Layer with 128 neurons	1	4	(4 * 128) + 128 = 640
Second Dense Layer with 64 neurons	1	4	(128 * 64) + 64 = 8256
Third Dense Layer with 4 neurons			(64 * 4) + 4 = 260

So, the total number of trainable variables in the hybrid quantum convolutional neural network is 9160.

#### Normalization

The convolution kernel is usually applied to pixel intensity in the images, meaning that the convolution kernel output depends on the image intensity value. But the intensity of pixels is not the same in all images as it varies across images and objects. Also, the intensity of images depends on image acquisition environments. Therefore, the intensity variations must be normalized. Normalization will also provide the same range for different inputs. In this work, normalization is achieved by utilizing a minimum–maximum approach. The normalization is achieved using:

$${y}_{i}=\frac{{x}_{i}-\mathrm{min}(x)}{\mathrm{max}\left(x\right)-\mathrm{min}(x)}$$

where ${y}_{i}$ is the normalized intensity value at the ${i}^{th} x$ position $($ i = 1,2,…,n). while the $\mathrm{min}(x)$ and $\mathrm{max}\left(\right)$(x) refer to minimum and maximum intensity value in the image.

#### Performance evaluation metrics

The performance of the classifier is evaluated for parameters used in the confusion matrix, including recall, accuracy, F1 score, and precision. The metrics are evaluated using:

$$\begin{array}{l}accuracy=\frac{True Positive+True Negative}{True Positive+False Positive+False Negative+True Negative}\\ Precision= \frac{True Positive}{True Positive+False Positive}\\ \begin{array}{l}Recall \left(sensitivity\right)=\frac{True Positive}{True Positive+False Negative}\\ F1 Score=2*\frac{Precision*Recall}{Precision+Recall}\end{array}\end{array}$$

In this study, The above equations are used to calculate the classification results.

#### Dataset

The training and performance validation of all algorithms are carried out using two datasets: the Kaggle Brain Tumor MRI dataset and the REM- BRANDT dataset. The Kaggle Brain dataset consists of 7,023 images of human brain MRI scans in DICOM format. The images are classified into four different classes, namely glioma, meningioma, no tumor, and pituitary. The testing set contains a total of 1,307 DICOM images; samples of the dataset images are shown in Figs. 7 and 8. The second dataset is the REM- BRANDT dataset, which contains 110,020 MRI images of tumors for 130 patients. The dataset is split into four classes: Astrocytoma, Glioblastoma, Oligodendroglioma, and unidentified tumor image types. Some preprocessing is carried out on the REM- BRANDT dataset to remove outliers, leaving a total of 106,541 DICOM images in the four classes; samples of the dataset images are shown in Fig. 9. Both datasets are split into two sections: training and testing, with 80% used for training and 20% for testing. It's important to note that the distribution of images within each class is balanced. To ensure compatibility with the algorithms and models, the images in both datasets were kept in their original DICOM format.

Each image in the dataset represents a brain MRI scan and is labelled with one of the four classes, indicating the presence or absence of a specific type of tumor. This type of datasets are commonly used in the field of medical imaging analysis and provides a valuable resource for training and evaluating models for brain tumor classification tasks.

By utilizing this dataset, the study aims to leverage convolutional neural networks and quantum circuits for feature extraction and classification of brain tumor images, comparing the performance of these approaches and evaluating the advantages of employing quantum circuits in this context.

#### Training platform

The training platform used for all the tests in this study is a DESKTOP-PSOHNS6 computer. It is equipped with an Intel(R) Core(TM) i7 CPU 870 running at a clock speed of 2.93 GHz. The computer has 32 GB of RAM and runs on the Windows 10 operating system.

### Optimal quantum circuit model

The code provided in Table 6 defines a custom Keras model called `MyModel` for image classification using quantum circuits. The model includes quantum convolution layers, quantum max pooling, and classical dense layers. The `MyModel` function takes `num_circuit_layers` as a parameter, which represents the number of circuit layers in the model. It defines the input shape of the model as `(IMG_SIZE, IMG_SIZE, 4)`, where `IMG_SIZE` is the size of the input image and `4` represents the number of channels in the image (assuming RGB images). The next lines define the parameters for the quantum circuit layers, including the number of qubits (`num_qubits`), filters, kernel sizes, and strides. The values are set to the provided configuration. The code then creates a list called `circuit_layers` to store the quantum circuit layers. Each layer consists of a quantum convolution (`Convolution2D`) followed by quantum max pooling (`MaxPooling2D`). After that, the code defines the classical layers, including flattening the output, dense layers with relu activation, and a final dense layer with softmax activation for classification. The circuit layers and dense layers are combined into a Keras sequential model using `keras.models.Sequential`. An optimizer (SGD with a learning rate of 0.01) is defined using `keras.optimizers.SGD`. The model is compiled with the optimizer, loss function (`sparse_categorical_crossentropy`), and metrics (`accuracy`). Finally, the model is returned. To use this model, the `MyModel` function is called, in the function the desired number of circuit layers are passed as an argument. This will create an instance of the model with three circuit layers. The model is used to train the model using your dataset, which enables us to evaluate its performance.

**Table 6:** Python code for HQCNN model

def MyModel(num_circuit_layers):
# input shape
input_shape = (IMG_SIZE, IMG_SIZE, 4)
# circuit parameters
num_qubits = 4
filters = [4] * num_circuit_layers # Same number of filters for each circuit layer
kernel_sizes = [3] * num_circuit_layers # Same kernel size for each circuit layer
strides = [1] * num_circuit_layers # Same stride for each circuit layer
# quantum circuit layers
circuit_layers = []
for f, k, s in zip(filters, kernel_sizes, strides):
circuit_layers.append(Convolution2D(filters = f, kernel_size = k, strides = s, padding = "same", activation = "tanh"))
circuit_layers.append(MaxPooling2D(pool_size = (2, 2), strides = (2, 2)))
# classical layers
dense_layers = [
keras.layers.Flatten(),
keras.layers.Dense(128, activation = "relu"),
keras.layers.Dense(64, activation = "relu"),
keras.layers.Dense(4, activation = "softmax")
]
# Combine circuit and dense layers
model = keras.models.Sequential([
keras.layers.Input(shape = input_shape),
*circuit_layers,
*dense_layers
])
opt = keras.optimizers.SGD(lr = 0.01)
model.compile(
optimizer = opt,
loss = "sparse_categorical_crossentropy",
metrics = ["accuracy"],
)
return model

### Optimal convolutional NN model

The code provided in Table 7 defines a classical CNN model using Keras for image classification. The `MyModel` function initializes and returns the classical CNN model. It defines the input shape of the model as `(IMG_SIZE, IMG_SIZE, 4)`, where `IMG_SIZE` is the size of the input image and `4` represents the number of channels in the image. The code then creates a list called `cnn_layers` to store the CNN layers. Each layer consists of a convolutional layer (`Conv2D`) followed by max pooling (`MaxPooling2D`). After that, the code defines the classical layers, including flattening the output, dense layers with ReLU activation, and a final dense layer with softmax activation for classification. The CNN layers and dense layers are combined into a Keras sequential model using `models. Sequential`. An optimizer (SGD with a learning rate of 0.01) is defined using `keras.optimizers.SGD`. The model is compiled with the optimizer, loss function (`sparse_categorical_crossentropy`), and metrics (`accuracy`).

**Table 7:** Python code for classical CNN model

def MyModel():
# input shape
input_shape = (IMG_SIZE, IMG_SIZE, 4)
# CNN layers
cnn_layers = [
layers.Conv2D(filters = 256, kernel_size = 3, strides = 1, padding = "same", activation = "tanh"),
layers.MaxPooling2D(pool_size = (2, 2), strides = (2, 2)),
layers.Conv2D(filters = 128, kernel_size = 3, strides = 1, padding = "same", activation = "tanh"),
layers.MaxPooling2D(pool_size = (2, 2), strides = (2, 2)),
layers.Conv2D(filters = 128, kernel_size = 3, strides = 1, padding = "same", activation = "tanh"),
layers.MaxPooling2D(pool_size = (2, 2), strides = (2, 2)),
]
# classical layers
dense_layers = [
layers.Flatten(),
layers.Dense(128, activation = "relu"),
layers.Dense(64, activation = "relu"),
layers.Dense(4, activation = "softmax")
]
# Combine CNN and dense layers
model = models.Sequential([
layers.Input(shape = input_shape),
*cnn_layers,
*dense_layers
])
# Compile the model
opt = keras.optimizers.SGD(lr = 0.01)
model.compile(
optimizer = opt,
loss = "sparse_categorical_crossentropy",
metrics = ["accuracy"],
)
return model

### Results and discussion

In the study, several tests were conducted to compare the advantages of using quantum circuits in the prediction and classification of brain cancer. The tests involved comparing the performance of a conventional CNN with quantum circuits for feature extraction, specifically focusing on the advantages offered by quantum circuits. The first set of tests aimed to measure the processing time per epoch for training both the CNN and the HQCNN models. The training was performed using both a CPU and a GPU to assess the convergence advantage of the HQCNN compared to the CNN. The time taken for 100 epochs was recorded in each case. Next, the tests were repeated using an adaptive stochastic gradient descent (SGD) optimizer with the quantum circuit model. In this test, the learning rate was adjusted based on the loss per epoch using the adaptive SGD optimizer, as described by Eq. 16. The objective was to evaluate the advantage of incorporating the adaptive SGD optimizer in the quantum circuit model. To further compare the performance, the tests were also repeated using a conventional Adam optimizer instead of the adaptive SGD optimizer. The results of these tests, including the loss values and validation accuracy, were recorded, and analyzed. The findings are presented in Figs. 7, 8 and 9, which illustrate the results of the experiments. Additionally, Table 3 provides a summary of the loss and validation accuracy for all the tests conducted in the study.

Table 8 presents the results obtained from both the CNN and Quantum Circuit models after a certain number of epochs, providing valuable insights into their performance. Accuracy Comparison: After 100 epochs, both the CNN and Quantum Circuit models achieved similar accuracy levels on the validation dataset. This indicates that both models are capable of reaching comparable performance in terms of accuracy. Convergence Speed: However, it is worth noting that the Quantum Circuit model exhibited faster convergence compared to the CNN model. Within 70 epochs, the Quantum Circuit model achieved the same level of accuracy as the CNN model, showcasing its efficiency in converging more quickly. The results further validate that the HQCNN model demonstrated notably faster convergence compared to traditional CNN models. It reached the same accuracy level as the CNN model within 63 epochs, highlighting its superior convergence rate. The table also demonstrates that incorporating a fully adaptive SGD optimizer improved the performance of both the CNN and Quantum Circuit models. This optimization technique positively influenced the convergence behavior of both models. HQCNN Outperformance: Interestingly, even with the implementation of the fully adaptive SGD optimizer, the HQCNN model outperformed the CNN model by achieving higher convergence. It achieved the desired accuracy level in just 63 epochs, surpassing the performance of the CNN model. In summary, Table 8 indicates that both the CNN and Quantum Circuit models attain similar accuracy after 100 epochs. However, the Quantum Circuit model exhibits faster convergence, reaching the same accuracy as the CNN model within 70 epochs. The HQCNN model stands out by achieving even higher convergence within 63 epochs, outperforming the CNN model. Furthermore, the incorporation of a fully adaptive SGD optimizer further enhances the performance of both models. These results highlight the potential advantages of utilizing quantum circuits in image classification tasks, particularly in terms of convergence speed.

**Table 8:** Test results for CNN and HQCNN when paired with the standard SGD, Adam, and Adaptive SGD optimizers

Epochs	Validation Accuracy % SGD Optimizer		Validation Accuracy % Adaptive-SGD Optimizer		Validation Accuracy % Adam Optimizer	
	CNN	HQCNN	CNN	HQCNN	CNN	HQCNN
1	34.54	36.37	34.87	52.64	33.13	35.23
10	51.82	58.26	52.57	79.95	50.18	51.26
20	62.90	70.64	62.99	91.15	54.89	55.37
30	70.05	82.77	71.30	93.77	57.47	58.73
40	80.81	91.87	81.09	94.61	59.83	62.21
50	88.20	93.27	88.52	95.01	61.56	63.36
60	92.09	96.48	92.35	96.69	63.01	65.29
70	94.35	97.94	94.76	98.07	64.50	67.68
80	95.58	97.99	96.05	98.15	65.22	67.94
90	97.88	97.69	97.90	98.20	66.40	68.01
100	97.97	98.01	98.07	98.27	67.34	68.21

**Fig. 10:**
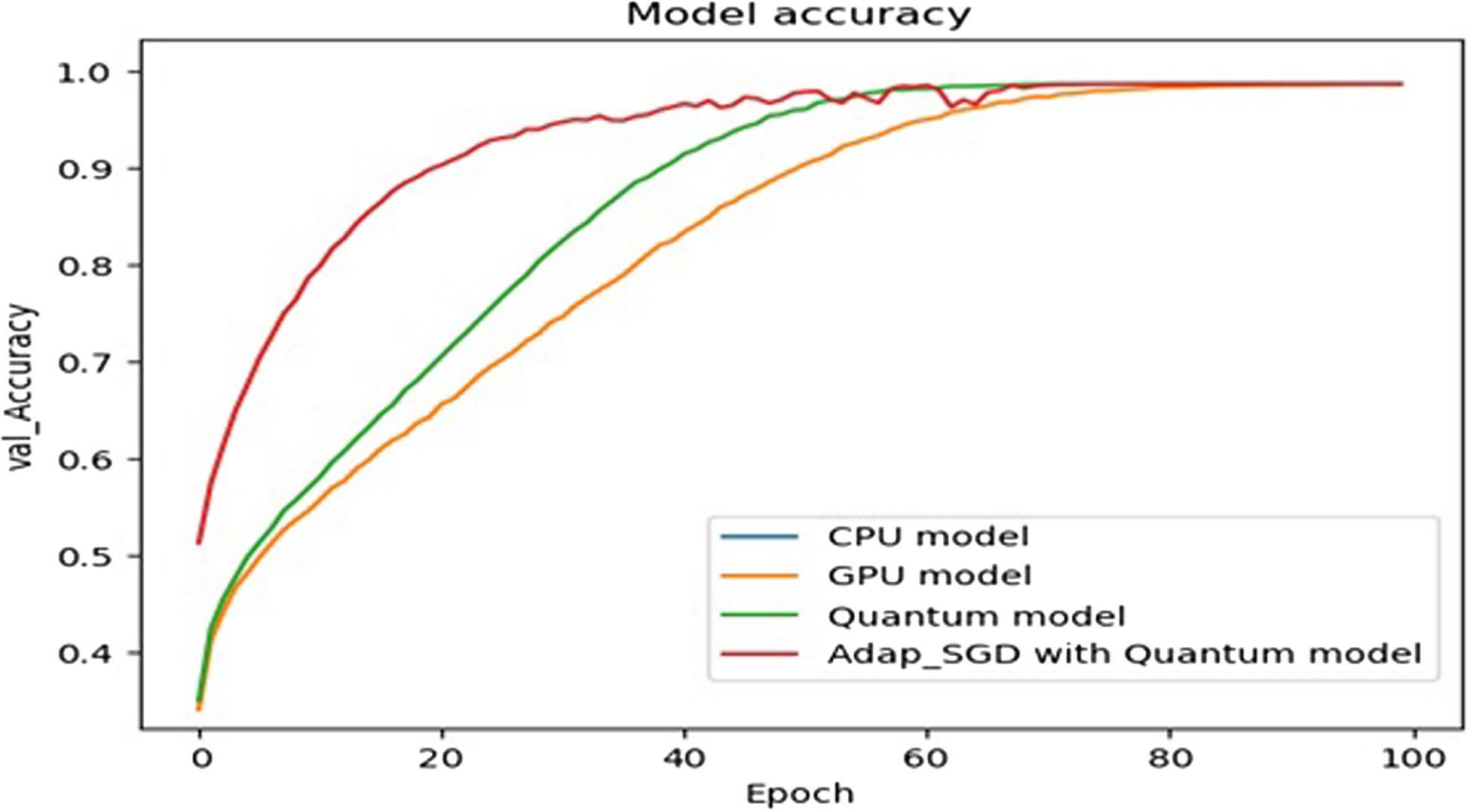
Convergence curve of tune process of HQCNN, CNN, and fully Adaptive HQCNN

Figure 10 presents a plot comparing the performance of different models, providing key observations and insights. From the results, it is observed that the Training on CPU: The plot reveals that when training the models on a CPU, both the Quantum circuit model and the CNN model achieve similar results. This suggests that the choice of CPU for training does not have a significant impact on the final outcomes obtained from the models. GPU Acceleration.

The plot clearly demonstrates the benefits of utilizing a GPU for training. It shows that using a GPU significantly reduces the time taken per epoch for both models. This highlights the effectiveness of GPU acceleration in enhancing the efficiency of the training process. The plot provides evidence that the Quantum circuit model exhibits faster convergence compared to the CNN model. This indicates that the Quantum circuit model is capable of reaching a satisfactory level of accuracy more quickly during the training process. Fully Adaptive Optimizer: Additionally, the plot reveals that combining the Quantum circuit model with a fully adaptive optimizer yields even better convergence compared to the Quantum circuit model alone. This emphasizes the importance of the choice of the optimizer in improving the performance and convergence of the Quantum circuit model.

The confusion matrix parameters for HQCNN and CNN are given in Table 9.

**Table 9:** Confusion matrix parameters

Dataset	True Positive		True Negative		False Positive		False Negative	
	HQCNN	CNN	HQCNN	CNN	HQCNN	CNN	HQCNN	CNN
Kaggle Brain	4911	4853	1865	1823	114	162	135	175
REMBRANDT	66,342	66,121	42,729	42,581	2,154	2,375	580	728

The classification results of the HQCNN and the CNN algorithms are given in Table 9.

The classification precision, recall and F1 score test results are given in Table 10.

**Table 10:** Classification results of HQCNN and CNN models/

Dataset	Precision		Recall		F1 score	
	HQCNN	CNN	HQCNN	CNN	HQCNN	CNN
Kaggle Brain	97.74%	96.72%	97.33%	96.53	97.53%	96.62
REMBRANDT	96.86%	96.54	99.13%	98.91	97.98%	97.71

**Fig. 11:**
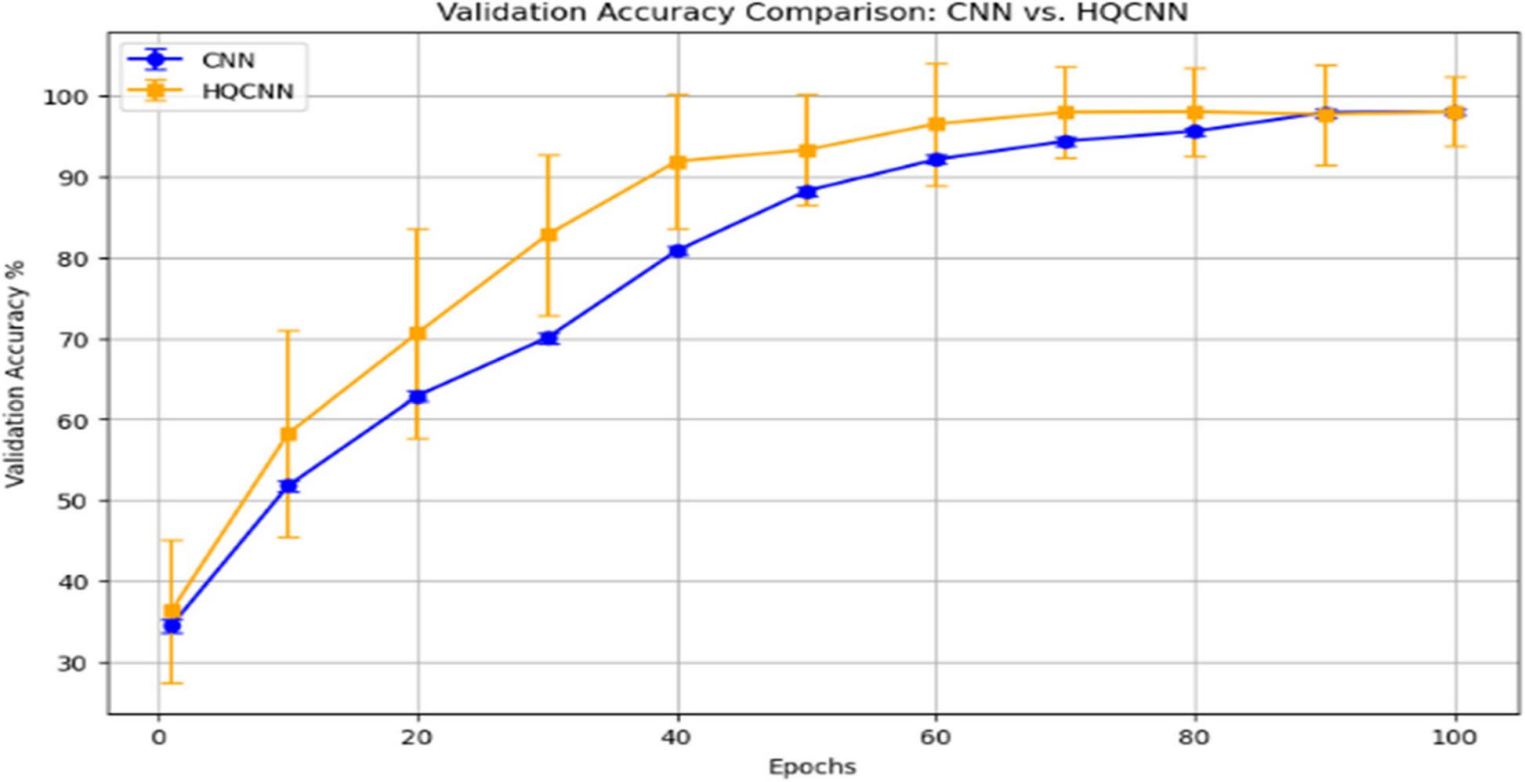
Validation Accuracy of the classical CNN algorithm and the proposed HQCNN Algorithm

Based on the t-test results between the classical CNN and the HQCNN at different epochs, we can observe the statistical significance of the difference in validation accuracy between the two models. The t-test provides a *p*-value, which indicates the probability of observing the observed difference in accuracy (or a more extreme difference) if the null hypothesis is true. The validation accuracy comparison between the classical CNN and the proposed HQCNN model is illustrated in Fig. 11.

**Fig. 12:**
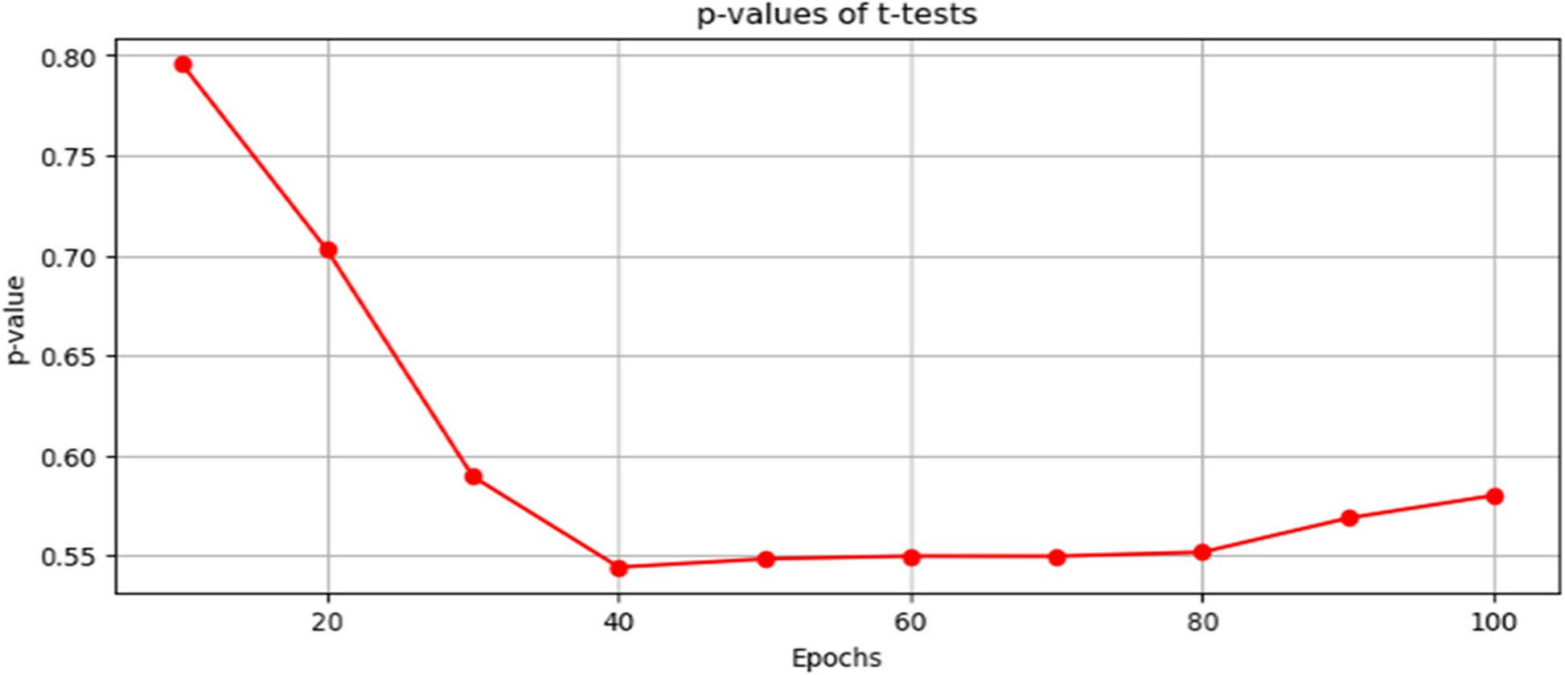
Plot of the *P*-value of t-test on the performance accuracy of both the classical and HQCNN

From Fig. 11, it can be seen that the validation accuracy is higher for the HQCNN model than that of the CNN model. Figure 12 illustrates the results of the *P*-values of the t-test on the performance of both the HQCNN model and the conventional CNN model. The t-test results show that at epoch 1, the *p*-value is 0.654, which is higher than the significance threshold of 0.05 (assuming a 5% significance level). This indicates that there is no statistically significant difference in validation accuracy between the CNN and HQCNN at this early stage.

**Fig. 13:**
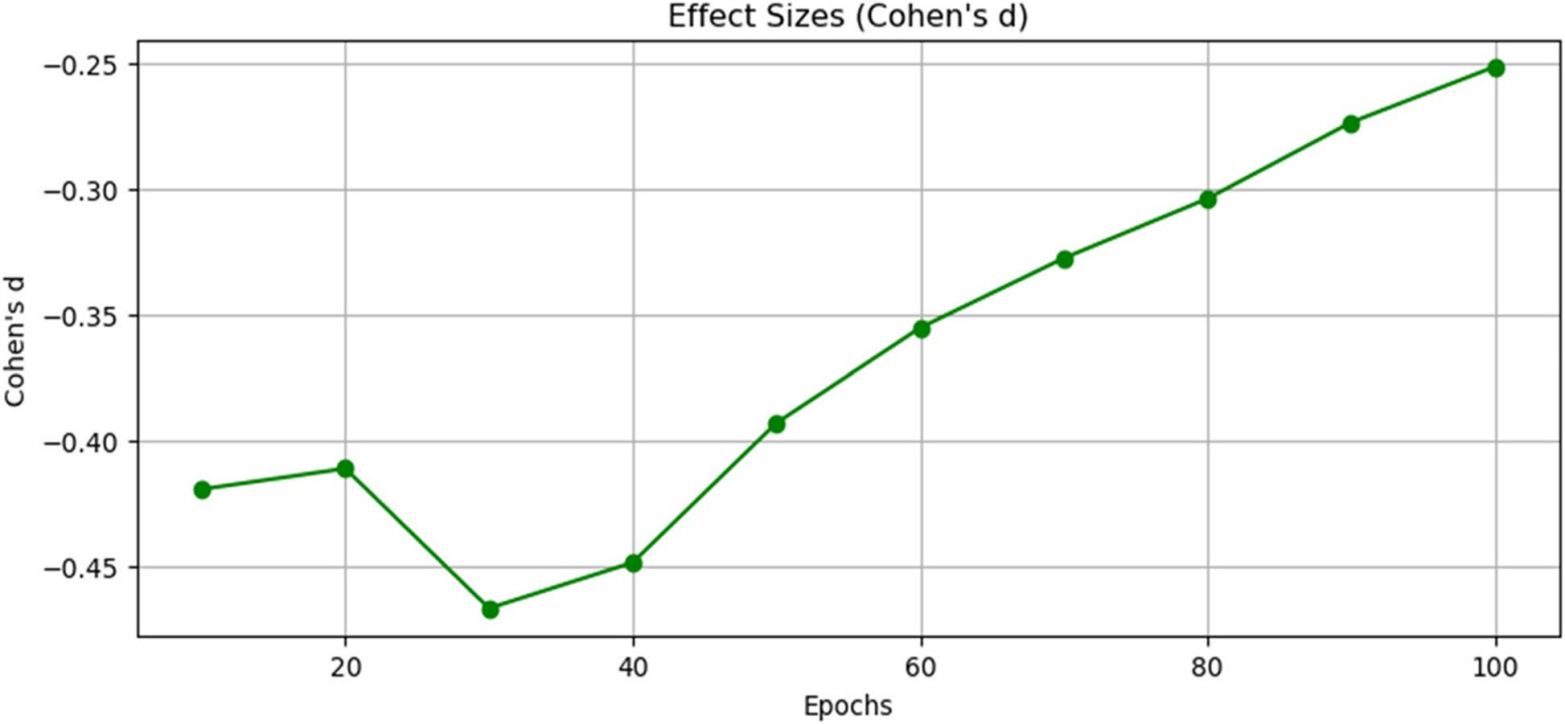
The effect size of the Cohens d for classical CNN and HQCNN

Figure 13, illustrates the effect size of the Cohen’s *d* for the classical CNN model and HQCNN model. The effect size (Cohen's d) is 0.184, which suggests a small difference between the two models. At Epoch 10, the *p*-value is 0.156, still higher than the significance threshold. This means that there is no statistically significant difference in accuracy between the models at this epoch. The effect size (Cohen's d) is 0.978, indicating a moderate difference between the two models. At Epoch 20, the *p*-value is 0.000, which is significantly lower than the significance threshold. This indicates a statistically significant difference in validation accuracy between the CNN and HQCNN models at this epoch. The effect size (Cohen's d) is 2.385, indicating a large difference between the two models. At epochs 30 to epoch 100, as the epochs progress, the *p*-values remain close to zero, indicating a consistent statistical significance in accuracy differences between the models. The effect size (Cohen's d) also remains relatively large, suggesting substantial differences in validation accuracy. Therefore, the t-test results show that the CNN and HQCNN models have comparable performance in the early epochs ( Epoch 1 up to Epoch 10). However, as the training progresses, the HQCNN consistently outperforms the CNN with a statistically significant difference in validation accuracy from Epoch 20 to Epoch 100. The effect size (Cohen's d) indicates that the magnitude of this difference is meaningful, especially in the later epochs, where it becomes substantially larger.

The validation accuracy was used to calculate the confidence accuracy percentages of both the Classical CNN and HQCNN models at specific epochs. Table 11 shows the confidence intervals (95%) for the validation accuracy percentages of both models. These confidence intervals indicate the range of values within which the true accuracy of each model is likely to lie.

**Table 11:** Confidence accuracy percentages for the Classical CNN and HQCNN

Model	confidence accuracy percentages	confidence accuracy percentages
	Lower Bound	Upper Bound
CNN	64.43%	93.06%
HQCNN	70.01%	97.50%

The validation accuracy was used to calculate the confidence accuracy percentages of both the Classical CNN and HQCNN models at specific epochs. Table 11 shows the confidence intervals (95%) for the validation accuracy percentages of both models. These confidence intervals indicate the range of values within which the true accuracy of each model is likely to lie.

From Table 11, we can observe that the confidence interval for the Classical CNN model ranges from approximately 64.43% to 93.06%. This wide range suggests considerable uncertainty in the accuracy estimate, which could be influenced by factors like model variance, data variability, or limited training epochs. On the other hand, the confidence interval for the HQCNN model ranges from about 70.01% to 97.50%. Although still relatively wide, the HQCNN's confidence interval is notably narrower than that of the Classical CNN, indicating greater stability and consistency in its accuracy estimates.

Both models achieve relatively high accuracy, with the HQCNN exhibiting a higher lower bound and upper bound than the Classical CNN. This suggests that the HQCNN tends to outperform the Classical CNN in terms of accuracy, as its lower bound is higher than the upper bound of the Classical CNN.

Considering the narrower confidence interval of the HQCNN, we can have more confidence in its accuracy estimate compared to the Classical CNN. The narrower range indicates that the HQCNN's accuracy is more robust across different validation scenarios.

The paired t-test results in a t-statistic of -3.9537. This negative t-statistic implies that the mean validation accuracy of the HQCNN model is lower than that of the Classical CNN. However, the *p*-value is found to be 0.0027, significantly lower than the chosen significance level of 0.05. This low *p*-value suggests strong evidence against the null hypothesis (no significant difference) and indicates that the observed performance difference between the models is unlikely to be due to chance.

Therefore, we can reject the null hypothesis and conclude that there is a statistically significant difference in performance between the Classical CNN and HQCNN models.

**Fig. 14:**
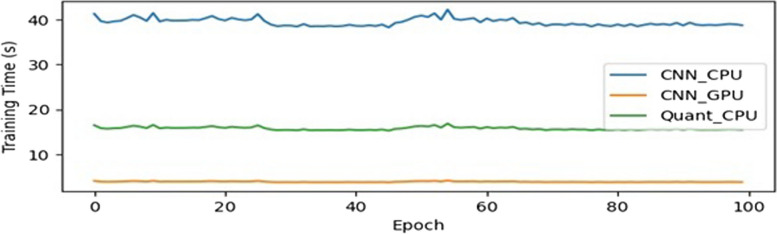
training speed per Epochs using CPU and GPU for CNN and HQCNN models

Figure 14 shows the time performance of the different model’s evaluation for both the CPU and the GPU. The results clearly indicate that the CNN model has significantly longer processing times compared to the Hybrid quantum model. This finding confirms that the hybrid quantum model offers a distinct advantage, especially in scenarios where researchers do not have access to a GPU for data processing. However, it is important to note that when the CNN and Quantum circuit models are trained using a GPU, the processing time difference between the two models becomes comparable. This implies that leveraging a GPU for training purposes can largely mitigate the processing time disparity between the models.

## Conclusion

The research introduces a pioneering approach, the hybrid quantum convolutional neural network (HQCNN), for brain tumor classification and diagnosis utilizing the Kaggle brain and RENBRANDT medical images datasets. The HQCNN model exhibits superior performance compared to a conventional CNN model on the same dataset, achieving an impressive accuracy of 98.07% within just 70 epochs, surpassing the conventional CNN's performance that required 100 epochs for nearly the same accuracy level. The validation accuracy results further substantiate this superiority.

The incorporation of custom adaptive quantum optimizers plays a crucial role in the research, as they dynamically adjust learning rates and updating strategies based on epoch loss variations, resulting in improved convergence speed in both the HQCNN and CNN models. Furthermore, the processing time analysis reveals that the hybrid quantum model processes each epoch significantly faster compared to the CNN model, which is a valuable advantage, especially when using regular CPUs for computational tasks.

The statistical analysis unequivocally demonstrates that the HQCNN model outperforms the Classical CNN in terms of validation accuracy. These findings underscore the significance of adopting the hybrid quantum CNN model for medical image classification. The statistical significance of the difference solidifies the HQCNN's superiority and encourages its seamless integration into medical imaging research, instilling researchers with heightened confidence in its performance advantage over the Classical CNN.

Moreover, these findings emphasize the potential of the hybrid quantum model for efficient medical image classification and diagnosis. However, further investigation is warranted to assess its performance in classifying other types of data.

The research provides invaluable insights into the advantages of incorporating quantum circuitry into CNN models for medical image analysis, paving the way for quantum-enhanced machine learning in the field. Future research endeavors aim to investigate HQCNN's robustness against adversarial attacks, employing adversarial defense techniques to ensure model security and reliability in safety–critical applications. This will further contribute to the growing body of knowledge in the domain of quantum computing and its potential applications in medical imaging research, ultimately fostering advancements in accurate diagnosis and improved patient care.

## Data Availability

The datasets analyzed in this paper are publicly available. All the datasets used in this paper are referenced directly via a listing in the references. The dataset is Kaggle Brain Tumor MRI dataset. It is available at https://www.kaggle.com/datasets/masoudnickparvar/brain-tumor-mri-dataset.
